# A Reference Hand-Book of the Medical Sciences

**Published:** 1887-04

**Authors:** 


A REFERENCE HAND-BOOK OF THE MEDICAL SCIENCES. Embracing the entire range of scientific and practical medicine and allied science. By various writers. Illustrated by chromolithographs and fine wood engravings. Edited by Albert H. Buck, M. D., New York city. Volume IV. Bound in cloth, sheep and half morocco. Prices, $6.00, $7,00 and $8.00 per Vol. New York: William Wood & Company.

This volume contains various practical subjects of the highest importance to every physician, and exceeds in some respects either of the three preceding volumes. All the contributions indicate careful preparation by their authors, and a number of them are illustrated in the best artistic style. The admirable colored plates of carcinoma and sarcoma of the larynx are faithful copies of the original forms of disease, while the elegant delineations of micro-organisms reproduced from Huber and Becker's work afford exquisite specimens of the combinations of colors by depicting the contents of the test-tube in the various stages of examining the cultures. Only a few of the numerous topics of special interest can receive attention in the brief notice of this volume which our space permits, but all its contents are valuable, and are commended to the careful attention of medical men who desire to keep abreast of the progress of medical science, not alone in the practical branches, but in any department of professional advancement.

Inflammation is considered chiefly as presented undr the microscope, with the most prominent theories in regard to its various processes by Councilman; but no allusion is made to Sutton's views

While in-growing toe-nail is not worthy of notice from the frequency of its occurrence, its consideration by Gay is confined to two columns. Insanity, in its various forms, is treated elaborately by Kellog, Bush, Davis and Stedman, occupying nearly a hundred pages. The intestines and their various acute and chronic disorders take up fifty pages under the authorship of Ross, Shepherd, Thompson, Bell, Dana, Cheeseman and Bryant, Much useful information is embodied in these papers, yet some points of practical consequence have been recently adopted which are overlooked by the writers. Jaundice, by Fitz; injuries and diseases of the jaws, by Briggs; injuries to the joints, by Bryant; each fill a few pages. The anatomy and affections of the kidney, with instructive illustrations, take up forty-four pages, contributed by Roosevelt, Mendelson, Baumgarten, Biggs, Hackley and Ransahoff. Labor, with all its complications, is exhaustively treated in forty-five pages by Withington, and is illustrated by numerous well-designed cuts. Laryngectomy, by Park; laryngoscope, with burns and other injuries of larynx, by Delavan; anatomy and physiology of larynx, by Westbrook; benign tumors of larynx, by Lefferts; carcinoma and sarcoma of larynx, by Morgan; catarrhal affections and syphilis of larynx, by Mackenzie; intubation of larynx, by O'Dwyer; stenosis of larynx, by Asch, and phthisis of larynx, by Bosworth, fill most profitably seventy pages, and meet fully the requirements on these topics.

An elaborate but brief contribution on ligature, by Senn, comes up to the measure of what should be expected on this important matter. Lithoploxy with lithotomy, by Cabot, and lithotomy, by Hingston, bring these up to date in most of the many improvements, but the use of the continuous current evacuator has escaped the attention of the last-named writer. The various affections of the liver are very well disposed of by Musser, Satterthwaite, French, Wendt, Allen, McArthur and Biggs; but there is no recognition of highly important practical measures presented by Harley and Willet at the last meeting of the British Medical Association, owing, perhaps, to the preparation of these papers at an earlier date. Lung troubles occupy twenty pages contributed by Cammann, Quinby, Herrick, McArthur and Manning. Twenty pages are devoted to massage by Graham. Mastoid operations, by Buck, sustains well the known practical discernment of its author, and will be read with profit. The concise paper on meningitis, by Whitaker, is commendable for its comprehensive brevity. Miasms is not relieved of thewill-o'-the-wisp surroundings, by Smart, nor are micro-organisms freed from mystery by Freeborn. The timely contribution on microscopy, by Wilkins, must close this partial list of contents, with the saving clause that much remains to be learned by a studious examination of these and other valuable papers in this volume, which is gotten up in the usual first-class style of the publishers.

